# Golf and Health, More than 18 Holes—A Bibliometric Analysis

**DOI:** 10.3390/healthcare10071322

**Published:** 2022-07-16

**Authors:** María del Mar Martín-García, José Luis Ruiz-Real, Juan Carlos Gázquez-Abad, Juan Uribe-Toril

**Affiliations:** Department of Economics and Business, University of Almería, 04120 Almería, Spain; jlruizreal@ual.es (J.L.R.-R.); jcgazque@ual.es (J.C.G.-A.); juribe@ual.es (J.U.-T.)

**Keywords:** golf, health, sport, social benefits, mental benefits, bibliometrics

## Abstract

Despite golf’s contribution to health, scientific production related to golf and health has been relatively scarce. This work aims to investigate the state of the art on golf and health and to identify existing gaps and the principal and most notable potential future research trends, contributing to connecting the reality of the facilities dedicated to the practice of this sport and its contribution to raising awareness of the importance of sport in maintaining health. A total of 179 articles were analyzed following the steps for systematic reviews and meta-analysis protocols based on the PRISMA 2020 methodology and QUORUM, and a bibliometric analysis was carried out. Research to date has mainly focused on the benefits of golf in improving health, preventing illness, slowing down aging, or as rehabilitation and on exploring the risks and injuries involved in playing golf. The different ways of promoting participation or changing the image of golf by showing its healthy side are outlined as research trends in the coming years. There is a lack of exploration of the use of technology, the effects of the sport on certain disorders related to psychosocial factors, and further knowledge of the relationships between playing intentions and health. This research provides essential information for researchers who plan to work with golf in the future.

## 1. Introduction

In 2016, following an absence of 112 years, golf was once again accepted as a bona fide sports discipline and returned to the Olympic Games [1]. This inclusion thus recognized a sport currently played by around 60 million people around the world [2]. According to The Royal and Ancient, the body that regulates the rules of golf globally [3], there are almost 40,000 facilities dedicated to this sport in 209 countries. Most of the world’s golfing facilities are located in North America (51%) and Europe (23%), followed by Asia (16%), Oceania (5%), and Africa and South America, both with 2% of the world’s supply of golf facilities [4].

With North America leading the way, both in terms of the number of facilities and players, its impact on the world economy is reflected in the total turnover of all activities related to this sport. In the USA, where 43% of world supply is concentrated, in 2019, it was valued at USD 84 billion [2]. The continued expansion of the sport is confirmed by the number of new golf courses worldwide, a total of 540 projects in 2020. Of these, 64% are associated with tourist developments, evidence of the close link between golf, tourism, and economic development. They are also proof of the expansion of this sport around the world [4].

Golf has been a relevant subject of study within a multitude of scientific disciplines [5]. Since 1987, the World Scientific Congress of Golf has gathered together researchers, industry professionals, and people interested in golf [6]. Sports as a source of physical activity and from the perspective of health sciences has become increasingly attractive to researchers in recent years. Specifically, golf as a physical activity and its related health benefits are two closely related aspects. The World Health Organization has stated that regular physical activity prevents non-communicable diseases and can improve mental health and quality of life. For this reason, it recommends regular physical exercise based on age and population groups [7]. However, more scientific evidence is needed of the beneficial and harmful effects of each of the sports disciplines that justifies the contribution of exercise to improving health in each sport [8].

Various studies point to the benefits of playing golf, including a reduction in stress, socialization [9], an improvement in physical health, and an increase in self-confidence and cognitive performance [10]. Those who play golf consider it a sport with an important social and psychological component [11] in which social and intergenerational connections are established [12]. However, its practice also carries related risks, including sun exposure, ball impact, or the possibility of injury. These injuries mainly affect the elbow, wrist, shoulder, and back, although most can be prevented, taking into account several factors, which can contribute to making golf a safe lifetime activity [13].

Scientific research to date has focused mainly on sports’ health benefits [14,15,16,17,18,19,20,21,22,23,24,25,26,27,28,29,30,31,32,33,34,35,36] and, more specifically, on golf [10,37,38,39,40,41,42,43,44,45,46,47,48,49,50,51,52,53,54,55,56,57,58,59,60,61,62,63,64,65,66,67,68,69,70,71,72,73,74] and the injuries and risks derived from the practice of this sport [13,75,76,77,78,79,80,81,82,83,84,85,86,87,88,89,90,91,92,93,94,95,96,97,98,99,100,101,102,103]. There is a need, therefore, to know what has yet to be analyzed as well as the new trends in research on golf from the perspective of the health sciences. In this sense, one of the new trends in research is the positioning of golf as a health sport [104], highlighting the importance of the golf–health binomial and the need to measure its evolution in scientific production. This study aims to investigate the state of the art on golf and health to identify existing gaps in the literature and the most notable trends for research in the coming years. Our goal is to synthesize the research conducted to date, evaluating and identifying the available published literature on the topic. This review aims to represent the most recent information available on the relationship between golf and health through a bibliometric analysis. The findings can serve as a starting point that connects the reality of the facilities dedicated to the practice of this sport and its contribution to raising awareness of the importance of sport in preserving health.

## 2. Methodology

A systematic review of the existing literature [105] was carried out to analyze the state of the art and trends in the published research on golf and health The review followed the normalized steps for systematic reviews [106] and meta-analysis protocols based on the PRISMA 2020 methodology [107] and QUORUM [108,109] and involved a bibliometric analysis. This methodology facilitates data classification through statistical techniques, including publications indexed in the primary databases, author affiliations, keywords, citations, and the topics that are of greatest interest to the scientific community [110]. Bibliometric analysis is being used with increasing frequency to gain a clear idea of the development of a scientific discipline or a topic of interest, providing a more objective approach than the traditional literature review [111].

The steps followed in carrying out the bibliometric analysis are detailed. First, the search parameters and the database of scientific publications were defined. The start year was 1970, and the end year was 2021. After several initial tests to assess the suitability of different keywords, the selected terms were “golf *”, “health *”, and “sport *”, since the primary purpose of this research was to find out the main trends about golf as a sport and how it related to health. The inclusion of the * allowed us to incorporate other words with the same root. The expression “sport” was included to avoid distortions regarding the aim of our study.

The online databases chosen to obtain the information were the Web of Science (WoS) Core Collection, a multidisciplinary database and a solid and reliable source of information, and Scopus. In addition, the WoS allows one to search and filter information using various bibliographic parameters and by breaking down the concurrence of specific terms.

The aforementioned search parameters yielded 169 publications in the WoS and 282 in Scopus. These initial results were then filtered by year and type of publication. The year 2022 was excluded, as the year had not concluded at the time of writing this paper. Only articles were selected, and proceedings and books were excluded as well as duplicate papers or those that did not correspond to scientific journals, obtaining a final tally of 131 records in the WoS and 282 in Scopus.

Additionally, a search was carried out in the PubMed database to verify that the articles found in this database were not present in the WoS or Scopus. A table is included as supplementary material showing a total of 628 articles found in PubMed; 79 were in the WoS or Scopus. Having confirmed that the information extracted was complete and correct, the data were exported to “.txt” format and then analyzed, one by one, according to bibliometric principles, refining the results and eliminating those that did not comply with the parameters established for the present investigation.

A systematic analysis was carried out on the 119 records, using the title, keywords, abstract, and, where necessary, the full article to confirm that the articles did indeed deal only with the issue of golf and its relationship with health.

Articles on local development, video games, or those in which the term golf was used as an example within a list of physical activities but whose main study aim did not coincide with those of our search, were discarded, resulting in a final sample of 102 articles.

**Figure 1:**
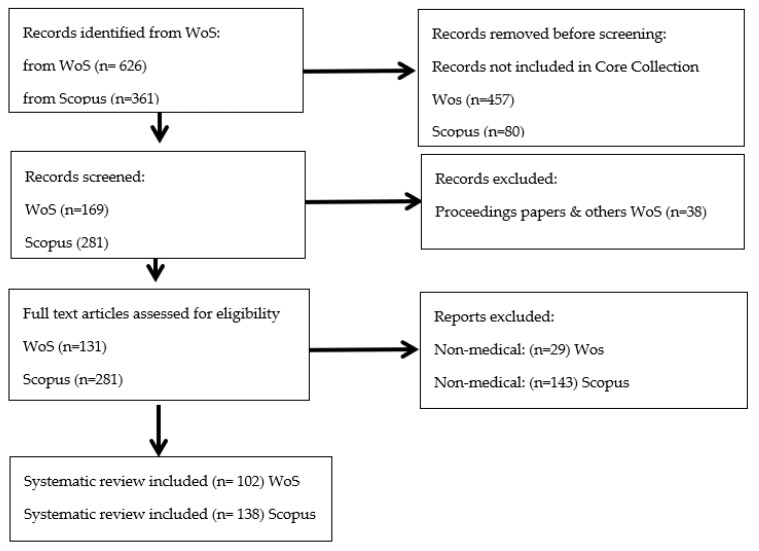
Flow diagram of systematic review (adapted from the Preferred Reporting Items for Systematic Reviews (PRISMA 2020) statement [108]).

Following this analysis, the final 102 articles in the WoS and 138 in Scopus were then analyzed for further discussion, as shown in Figure 1. To this end, different factors were considered, such as the annual evolution of scientific production, the most relevant countries, the most influential authors, and the most relevant journals in this field. The bibliometric analysis considered two factors: the volume of articles published on a specific topic and the citations that these works received, since both indicators provide valuable information on the result of the research [112]. Thus, bibliometric indicators such as impact (or the number of citations) and frequency serve to analyze the research situation on the topic. To analyze the impact of an author, the Hirsch index (h-index), which considers productivity and citation impact on the same result in a balanced way, was used. Finally, the most significant recent research trends were also identified.

The h-index is an indicator that evaluates the cumulative impact of an author’s scholarly output and performance. This metric takes stock, measuring quantity with quality, by comparing publications to citations. The h-index quantifies scientific productivity based on the authors’ publication histories [113].

Finally, for a more detailed analysis of the authors’ keywords, graphical representations in the form of cluster maps were developed using the VOSviewer software version 1.6.9. We only used this software for network analyses performed with the co-occurrence of “All Keywords” and following a fractional counting. The minimum number of occurrences of a keyword was 2; we found 95 different keywords. Likewise, cluster maps were developed to group certain key terms through co-occurrence analysis using network mapping techniques.

## 3. Results and Discussion

### 3.1. Annual Scientific Production per Year

**Figure 2:**
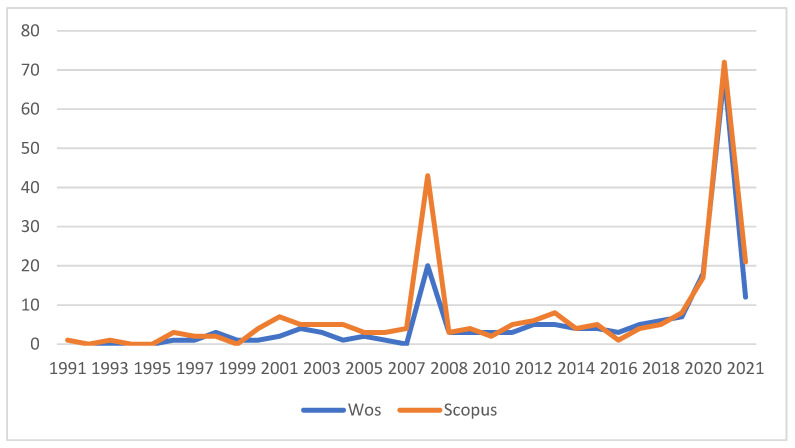
Number of articles per year.

Figure 2 shows the publication of articles per annum since 1991. Initially, production was scarce. Before 1996, we found one article that was published in both databases in which physical activity was quantified in a cohort of adolescents in New Zealand, pointing to golf as one of the most frequently played sports [114]. This article stands out, in addition to being the first, for its impact, since it received 49 citations. In 1993, only in Scopus, we found an article dedicated to injuries during golf practice, with a study of three cases [89]. Between 1996 and 2007, 49 articles were published, with an average of just below five articles published per year.

**Figure 3:**
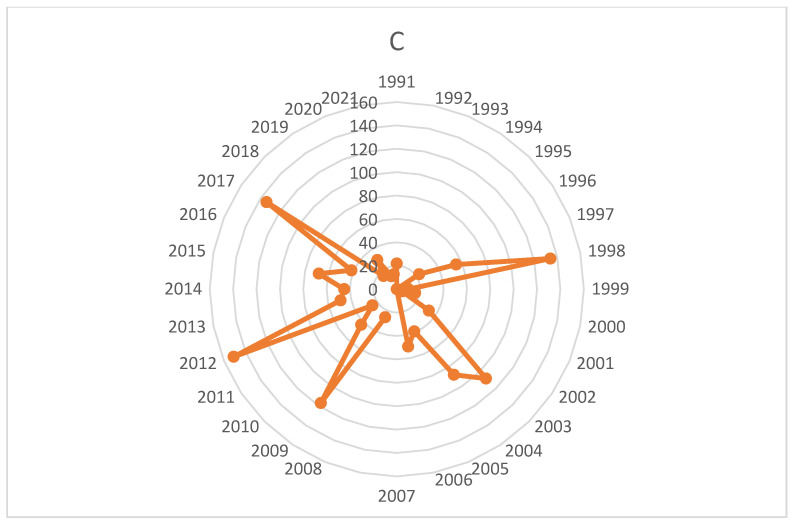
Number of citations per year (WoS).

Despite a low level of scientific production on this topic, as shown in Figure 3, it was during this period that the most cited articles to date were published. In 1998, the article concerning injuries resulting from playing golf [13] had the most significant impact to date, with 108 citations. Interestingly, this article did not appear in our search on Scopus, because it was classified as a review. This article described the most common golf injuries, their causes, and a way to prevent them. Fifteen more articles focusing on golf injuries [77,78,79,80,81,91,92,93,94,95,96,97,98] and how to prevent them [90] were also published during this period; of these, five were in both databases [77,78,79,80,81], and we found one piece of research on a device for relieving pain while playing golf [115]. Finally, we found five articles on the prevention of risks on the golf course during the practice of the sport [116,117,118,119,120] and an article on disease and sports, including golf [121].

Thus begins the scientific community’s interest in preventing aging through sports, which continues to this day. The benefit of sports in general [31], and golf in particular, in preventing non-communicable diseases was investigated in a total of 17 articles. These studies focused on the benefits of playing golf [42,43,44,48,49,50,51,52,53,54], the benefits of sport in general for cardiovascular health [24], the benefits of playing golf for the elderly [21,39,55,56], and the benefits of sport in preventing childhood obesity [22], matching three articles in both databases [21,42,43,44]. It is worth mentioning that the study published in 2004, with an impact of 84 citations, also identified the motivations of Australians over 55 years of age to participate in sports [21]. Furthermore, some studies also focused on particular techniques or technology used to increase golf performance during this same period [122,123,124], matching in both databases.

Finally, four papers were published in different fields of study. One of the articles with the most impact to date, published in 2003 with 82 citations, was the research on the return to sport after surgery in young athletes [125]. Another article, which garnered 54 citations, dealt with doping in sports [126]. The remaining two articles focused on alcohol [127] and focal dystonia [128] in professional golfers, respectively. A further three articles covered various topics: the evaluation of medical services in the professional golf circuit [129]; the control of movement in exercise [130]; and social interaction [131]. Three of the articles could be found in both databases [125,126,127].

Starting in 2008, an average production of almost six articles per year began to be the norm. Since 2017, production has increased. A total of 100 articles were published from 2008 to 2020 that relate golf and health. Of these 100 publications, 41 matched both databases [10,15,16,19,20,23,25,26,27,28,30,37,40,41,46,47,76,82,83,84,88,104,132,133,134,135,136,137,138,139,140,141,142,143,144,145,146,147,148,149,150].

The scientific production in these years dealt with various concepts. The central line of research focused on the benefits of sport as a source of physical activity for health, with 46 publications. This is the theme which has most grown in importance within this academic field and in which we found studies that analyzed sports in general, including golf.

Among the 24 works were studies related to living a healthy lifestyle in the Czech Republic [25]; physical activity to reduce mortality [16,17,18,19,151,152,153]; or rehabilitation after overcoming certain diseases [14,26,27,154,155,156,157,158,159]. The works dedicated to the benefit of sport in the elderly stand out [15,17,18,20,23,28,29,30]. It should be noted that one of the articles with the most significant impact, published in 2012 and with 88 citations, focused on the prevention of falls through physical activity in a group of elderly Australians [23]. Ten publications coincided in both databases [15,16,19,20,23,25,26,27,28,30].

This series of articles was completed with those dealing with the specific benefits of golf for health [31,32,34,57,58,59,60,61,62,63,64,65,66,67,68,69,70], particularly in the elderly age group [10,39,41,46,47]. Six publications could be found in both databases [10,37,40,41,46,47].

The next line of research in importance by publication volume was the study on injuries and risks in the practice of sport, with a total of 21 works. Scientific interest in this particular trend increased in this period but not as much as the main line. Of the 21 works dealing with this topic, 13 focused exclusively on golf, while the remaining 8 studied a range of sports (including golf) and analyzed the risks of outdoor sports [132,160] and injuries caused while engaged in sports [87,133,134,135,161,162]. Among the group of studies focusing exclusively on golf, there were studies on injuries among golfers [75,82,85,159,163,164,165] and the possible risks for players on the golf course [76,83,84,85,86,88,166]. In this line of research, we found nine publications in both databases [76,82,83,84,88,132,133,134,135].

Since 2008, eight articles have been published on different techniques, including technology, in research in sports and health [136,137,138,139,140,167,168,169]. Two studies, both published in 2017, are worthy of note. The first study dealt with the method of thinking aloud, with 42 citations [136]. The second study analyzed a device used to monitor health in sports and obtained 34 citations [137]. Five of these publications were in both databases [136,137,138,139,140].

Another line of research was the study of various disorders or diseases in athletes [141,142,170,171] or golfers [143,144,172,173,174,175,176]; body composition and nutrition in athletes [145,146,177]; the measurement of physical activity in adolescents [178]; and the use of alcohol in sports and the attempt to change behavior and culture [179]. Finally, there was one research work that tested the reliability of a health questionnaire among golfers [180]. Six of these investigations coincided in both databases [141,142,143,144,145,146].

Since 2008, particularly between 2011 and 2015, another emerging line of research has been the analysis of various ways of promoting participation in sports due to its contribution to health [147,148,181,182]. Three of these articles were in the two databases [147,148,182].

More recently, in 2019 and 2020, there was growing interest, reflected in the studies by Huth and Breitbarth, on the future role of golf as a health sport [104] and in marketing strategies for positioning golf in the market [11]. Only one of these articles was in both databases [104].

Additionally, in 2020, in two separate studies, Murray and Hawkes, together with other authors, investigated ways to evaluate and maximize the impacts of research on golf and health [149] and the methodology used for an International Consensus statement on golf and health [150]. Both articles were in the two databases.

Already in 2021, we found a large number of publications, as in 2020, specifically, 28 publications that related golf and health [32,33,34,35,36,71,72,73,74,99,100,101,102,103,183,184,185,186,187,188,189,190,191,192,193,194,195,196,197,198], 6 of them in both databases [71,73,100,102,185,190]. The main line of research continues to be the health benefits of sport [38,39,40,41,42] and studies on injuries [145,146,177,178,179] or risks to players on the golf course [183,187].

Unsurprisingly, publications dedicated to the COVID-19 pandemic [185,186,190,192] were included. The rest were publications on the return to sport after an intervention [189,193,196]; the use of technology to increase performance in the game of golf [191,194,197]; and one publication on the health effects of alcohol consumption on golfers [184].

### 3.2. Most Influential Countries

Regarding the publications that we found in the WoS, the 18 countries with the highest number of published articles are shown in Table 1. American authors participated in 40 articles, more than 40% of golf and health articles. The USA, along with Australia, is the leading country in terms of financing studies and scientific production on this subject. The lines of research that have been financed related sport with cardiovascular health and a reduction in mortality [16,19,24]. There were also two other studies on brain health and sports with a risk of impact [134,135] and two studies on educating for sports [148] and the role of golf as a means of rehabilitation for amputees [140].

The countries that follow the USA in the number of published articles, at a considerable distance, are the United Kingdom and Australia, with 21 and 15 articles, respectively. Likewise, in Australia, different organizations and institutions funded seven studies, five of them on the benefits of sport as a source of physical activity for the prevention of falls and diseases in elderly Australians [17,20,23,30,39], in addition to other studies along the same line but in the context of adolescents [178], and a study on the relationship between sport and alcohol which analyzed behavior in sports clubs concerning alcohol [179]. As for the United Kingdom, two of its publications have had the most impact to date among the articles that related golf with health [125,126,127,128,132,133,134,135,136,160,161].

The abovementioned three countries are followed by Germany and Canada, with 13 and 9 articles, respectively, and Japan, New Zealand, and South Korea, with 3 to 6 articles. Finally, the remaining countries that appear in the table have fewer publications, the majority with only one published article.

The 20 countries with the highest number of published articles in Scopus are shown in Table 1. The ranking of countries with the most publications is the same in the first six positions, with slight variations in the countries that follow them in publications with respect to the WoS.

### 3.3. Most Relevant Journals and Authors

No single journal contained a majority of articles. As shown in Table 2, the journal with the highest number of articles, according the WoS, is *Qualitative Research in Sport Exercise and Health*, which focuses on qualitative research in sport and exercise. This seems logical, since, as we have analyzed, the leading topic of scientific interest was related to the benefits of sport in general and, more specifically, golf in preserving health through physical exercise. Also noteworthy is *The Journal of Aging and Physical Activity*, according to the findings of this research, in which we found 48 publications dedicated to the benefits of golf in the fight against aging. It is followed by journals such as *The Journal of Science and Medicine in Sport* (Australia) and *Qualitative Research in Sport Exercise and Health*, dedicated to sports medicine, physical effort, exercise, and sports science.

Regarding the ranking of journals in Scopus, as can also be seen in Table 2, *The International Journal of Environmental Research and Public Health* stands out by number of publications; it is a journal dedicated to the interrelationships between environmental health and quality of life which links several scientific disciplines. This shows, as we have already pointed out, that golf has been a relevant subject of study within a multitude of scientific disciplines.

Table 3 shows the most cited articles in the WoS. These papers reflect the impact of the two main lines of research identified. The most cited article to date, “Golf injuries—An overview”, as mentioned above, was one dealing with golf injuries. Three of the most cited articles were identified with the main line of research found: the health benefits of sport in general, in the elderly population, and as rehabilitation after surgery. Finally, an article based on narrative theory which explored how the stories that an athlete told throughout her life in sport affected her career transition experiences was one of the publications with the highest impact. Interestingly, the most recent of these was from 2012. Since this time, no article has achieved as much impact to date.

Additionally, we include as supplementary material a table with the ranking of authors by number of publications in the WoS and Scopus.

Table 4 lists the top-ranked affiliate institutions associated with the scientific production on the subject of golf and health in both databases. According to our analysis, these institutions are in the countries that publish the highest number of works, Australia, the USA, Canada, and Germany. Edinburgh enters the ranking thanks to two works published in 2021 [185,190]. The entry of institutions from South Korea or China is due to work related to virtual golf [186,187] or the use of technology in sport [194]. However, despite the USA being ranked among the top countries in production volume, no institution stands out.

Regarding funding entities, Table 5 shows the top six entities that financed at least two research studies on the subject of golf and health. The first entity is an Australian funding body that sponsors medical research which funded four studies on the benefits of sport in preventing diseases in the elderly [17,20,23,30].

Together with the Department of Industry Innovation and Science, the Australian Government financed two studies, one on the subject of benefits and motivations of the elderly in Australia for playing golf [39] and the other on motor skills in adolescents [178].

Australia has one of the highest life expectancies globally, which may explain its academic interest in the benefits of sport in improving the health of its older population. Thus, through different organizations, this country, together with the USA, has financed the most studies related to sports and health. Its interest in the development of science and research is shown in the investment made by one of the organizations dedicated to health research, the National Health and Medical Research Council of Australia. In 2019, it granted up to AUD 4 million in funding to support Australian researchers [199].

The remaining entities that financed at least two works are in the USA. The National Institutes of Health, NIH USA, is the primary agency of the United States government responsible for biomedical and public health research and has financed three works together with the United States Department of Health and Human Services on the benefits of sports in preventing cardiovascular diseases [16,19,24]. The National Heart Lung Blood Institute, NHLBI, also participated in funding two of these studies [16,19].

The US National Institutes of Health is a medical research agency that annually invests more than USD 32 billion in research to improve health and reduce disease [200], which may explain the funding of these works dedicated to the prevention of cardiovascular diseases.

### 3.4. Most Frequently Used Terms and Main Trends

As shown in Table 6, our analysis found eight keyword terms that appeared a minimum of 10 times. It seems logical that the most used terms were “golf”, “health”, “sport”, and “exercise”. The most frequently used term, by a long margin, was “physical activity”, which is understood as the term “sport” or “exercise” in its broadest sense and is consistent with the main line of research found, that of the benefits of sport (again, “physical activity” in its broadest sense) for health. The eighth most widely used term was “injuries”, a finding consistent with this being the second most widely published topic.

**Figure 4:**
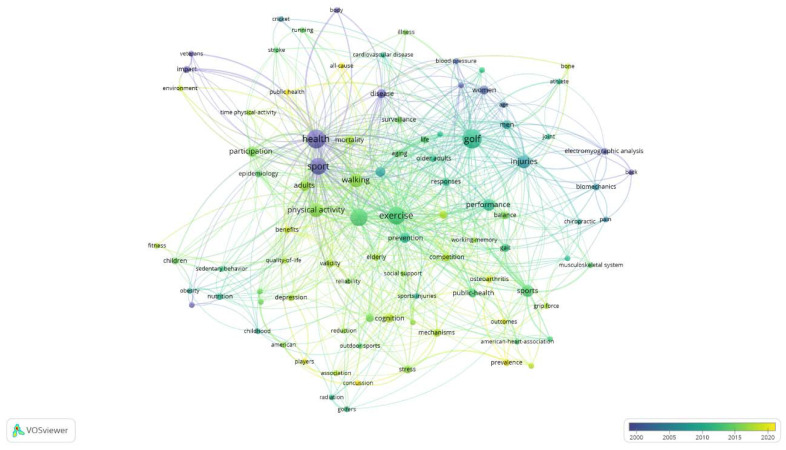
Temporal distribution of keywords (WoS).

Figure 4 indicates that older concepts were related to sports injuries and their relationship to performance and rehabilitation. In contrast, the concepts studied more recently were related to the benefits of physical activity as a source of health through sport in general and golf in particular. The keywords related to this concept (in green and yellow) were “walking”, “physical activity”, or “mortality”. Terms such as “participation” and “public health” (in yellow) revealed the latest trend, focused on studying different ways to promote participation in sport and the positioning of golf as a health sport.

The trends identified are consistent with the importance that leading a healthy life has acquired today, with physical activity being considered essential for disease prevention. In the specific case of golf, it acquires more importance, as it is a sport that provides a psychosocial component, played by all age ranges and contributing to good physical and psychological health, and consistent with the European Union’s concern about health for all [201].

The work framed within the Golf and Health Project, promoted by two organizations of international importance in the world of golf—The Royal and Ancient and the World Golf Foundation [149,150]—is striking. The project aims to raise awareness among the general public and those responsible in public institutions about the health benefits of golf. This work supports the principle that golf can generate more significant benefits for the health and wellbeing of both participants and spectators of golfing events. Therefore, it is intuited that this will be one of the main trends in research in the nearest future.

## 4. Discussion

Despite the importance currently placed on health and sport as a tool for achieving wellbeing, scientific production related to golf and health has been relatively scarce. Until 2020, there has not been a substantial increase in publications, a trend that is expected to continue.

The USA is by far the most prolific country in terms of articles published on this subject. This is not surprising, since it is the top-ranking country globally in the number of players and golfing facilities. In 2020, in the USA, there were 36.9 million Americans both on- and off-course [202]. However, despite its high volume of scientific production, no country rises above the rest in terms of impact.

The main line of research analyzed the benefits of sport in general and golf in particular in preventing diseases, especially in the elderly population, and maintaining good physical and mental health. Of the 179 articles identified in this study, 71 dealt with this topic.

Australia, the third-ranked country in the number of articles published, stands out in this line of research. Together with New Zealand, it contains 96% of the golf courses in Oceania, where golf is a very popular activity, largely due to its British influence [4], which may explain the interest in this subject. With only four articles, New Zealand has the second most impactful study to date, which may explain why it is the country with the most citations per article.

For the United Kingdom, a country with a long golfing tradition, it is logical that it is the second country in terms of scientific publication on the subject of golf and health. It is the leading European country in terms of number of players and the country where the game of golf originated before being exported to the rest of the world.

The rest of the countries with the most publications, Germany, Canada, Japan, and South Korea, are also the countries with the highest number of golf facilities, which may explain the interest in the relationship between golf and health. Indeed, 78% of the world’s supply of golf courses is concentrated in ten countries: the USA, Japan, Canada, the UK, Australia, Germany, France, the Republic of Korea, Sweden, and Scotland [2].

The second most discussed topic was injuries and risks in sports in general and golf in particular. Along these lines, the most cited article, “Golf injuries—An overview” [13] by Thériault, on golf injuries, stands out. It is striking that this Canadian author has only published this article on golf and health.

The most cited articles originated in the countries with the most publications, Canada [13], New Zealand [21,22], Australia [23,174], England [125,136], Germany [126], and China [142]. Although not among the countries with the most publications, the latter stands out, with 47 citations from a single article.

The above notwithstanding, the most cited articles did not have a common denominator, dealing with a broad range of topics, and were published by different authors and countries.

The relationship between golf and health is transversal, and articles about this topic were, thus, published in a broad range of journals. There was no concentration of articles in a single journal.

This scenario was repeated in the case of the authors. That is, they did not favor any particular journal. It is striking that, despite the USA being the country that contributes the most in terms of publications, no American author stands out by number of publications.

Concerning the principal affiliated institutions, these corresponded to the United Kingdom, Australia, the USA, Canada, and Germany, which are also among the countries with the highest production volume. The University of Edinburgh stands out, coming from the second country in terms of publications, the UK. In the case of the USA, despite being the country showing much difference with respect to the others and a leader in publications, we only found one institution, the University of North Carolina, to point out.

Regarding the entities that have financed research, Australia and the USA once more stand out. The case of Australia is significant, since almost half of the publications received financial backing.

In the analysis of the most relevant countries and institutions committed to research in this field, an absence of scientific production was observed in European countries which have well-established golf tourist destinations yet show little interest in research on golf and health. Such is the case of countries such as Spain, Portugal, or Italy. The case of Spain is particularly noteworthy, as it does play an essential role in research in other related areas, such as tourism marketing.

Regarding the keywords analyzed, the analysis showed an evolution in the lines of research, from articles on the subject of sports injuries and risks towards research associated with the benefits of playing sports, focusing on the prevention of aging and non-communicable diseases.

This evolution gives us a clue as to where research is likely to focus in the next few years. As pointed out, in 2020, a new line of research emerged related to the positioning of golf as a health sport. It is positioned to become the foremost academic trend in the coming years. In this sense, there is a lack of studies that can reaffirm the repositioning of golf as a health sport, linking health motivation with intentions to play golf.

In 2018, an international consensus was established on the relationship between golf and health, determining that playing golf regularly was associated with increased longevity, improved risk factors for cardiovascular disease, increased mental wellbeing, and the ability to positively influence the health of people with disabilities and contribute to active and healthy aging [150].

Thus, golf, as a sport that is practiced from childhood through to advanced ages, can help improve health and wellbeing throughout the life of the individual [39].

However, given the psychosocial component that golf has, there is a lack of research that relates this sport and its possible beneficial or harmful effects with certain diseases that this psychosocial factor can influence, such as Parkinson’s or Alzheimer’s.

The paucity of research on the use of health information devices that improve sporting skills and may help prevent injuries in golfers is striking. We found only two studies [137,138,139] on the use of this technology.

This research provides essential information for researchers planning to work with golf in the future. It points to the important contribution of the sport to health, with almost 40% of the research identified covering this topic, and will help to guide future research based on the gaps identified.

## 5. Conclusions

A systematic review identifies gaps, deficiencies, and the main lines of current research to help guide future research in a given area [203]. Two main lines of research have been highlighted in this paper, one which evidences the health benefits of golf and one which explores the risks and injuries involved in playing the sport. It is expected that future research will follow several trends, the main one being one that positions golf as a health sport. However, studies linking health motivation to intentions to play golf are still lacking. Several avenues of research are open, such as the use of technology to help to prevent golf injuries or the study of the effects of golf on certain disorders with an important psychosocial factor, such as Parkinson’s or Alzheimer’s disease.

This study is not exempt from limitations. However, we believe that, with respect to the criteria for selecting keywords, including the main keywords for our research, it might be interesting to include more keywords or other databases in future research.

## Figures and Tables

**Table 1:** Countries by number of publications (Scopus and WoS).

R	Country Scopus	N.A. Scopus	Country WoS	N.A. WoS	Citations WoS	TC/Art WoS	h-Index WoS
1	USA	51	USA	40	372	9.30	10
2	UK	21	UK	21	375	17.86	8
3	AUSTRALIA	23	AUSTRALIA	15	232	15.47	8
4	GERMANY	13	GERMANY	13	97	7.46	5
5	CANADA	10	CANADA	9	180	20.00	5
6	JAPAN	8	JAPAN	6	57	3.00	3
7	CHINA	1	NEW ZEALAND	4	217	54.25	4
8	CZECH REPUBLIC	2	SWITZERLAND	4	20	5.00	2
9	SOUTH KOREA	6	SOUTH KOREA	4	5	1.25	1
10	FRANCE	3	SPAIN	3	15	5.00	2
11	HONG KONG	4	PEOPLE R CHINA	2	60	30.00	2
12	NEW ZELAND	3	FRANCE	2	35	17.50	2
13	SOUTH AFRICA	1	TAIWAN	2	15	7.50	1
14	SPAIN	1	SLOVENIA	1	48	48.00	1
15	SWIZERLAND	4	NETHERLANDS	1	23	23.00	1
16	TAIWAN	1	BRAZIL	1	13	13.00	1
17	BRAZIL	2	NORWAY	1	4	4.00	1
18	NORWAY	2	CZECH REPUBLIC	1	2	2.00	1
19	AUSTRIA	1					
20	DENMARK	1					

R: Ranking; N.A.: number of articles TC/Art: total citations per article; h-index: Hirsch index.

**Table 2:** Most relevant journals (WoS and Scopus).

R	Journal Scopus	IFS	AS	Journal WoS	IFW	AW	CW	TC/A W	h-i W
1	Int. J. Environ Res. Public Health	0.743	7	Br. J. Sports Med.	13.8 (Q1)	2	5	2.50	1
2	J. Sci. Med. Sport	1.575	6	Sports Medicine	11.14 (Q1)	2	117	58.50	2
3	J. Aging Phys Act.	0.684	4	Qual Res. Sport Exerc.	6.736 (Q1)	5	206	41.20	4
4	Am. J. Sports Med.	3.021	3	Med. Sci. Sports Exerc.	5.411 (Q1)	3	29	9.67	3
5	BMJ Open Sport Exerc. Med.	1.724	3	J. Sci. Med. Sport	4.319 (Q1)	4	72	18.00	3
6	Br. J. Sports Med.	4.329	3	Sports Health	3.843 (Q2)	2	25	12.50	2
7	J. Manipulative Physiol. Ther.	0.448	3	J. Athl. Train	2.86 (Q2)	2	31	15.50	2
8	Qual. Res. Sport Exerc. Health	1.482	3	Sociol. Sport J.	2.134 (Q3)	2	12	6.00	2
9	Ger. J. Exerc. Sport	0.447	2	J. Aging Phys. Act	1.961 (Q3)	4	98	24.50	3
10	Harvard Men S. Health Watch	0.113	2	J. Manipulative Physiol. Ther.	1.437 (Q3)	2	6	3.00	2
11	J. Arthroplasty	2.766	2	Phys. Cult Sport Stud.	n.i.	3	3	1.00	1
12	J. Bone Joint Surg. Am.	2.634	2	BMJ Open Sport Exerc. Med.	n.i.	2	33	16.50	1
14	J. Hum. Sport Exerc.	0.913	2	Ger. J. Exerc. Sport	n.i.	2	55	27.50	1
15	J. Orthop. Sports Phys. Ther.	1.367	2	Int. J. Sports. Phys. Ther.	n.i.	2	30	15.00	2

R: Ranking; IFS: impact factor Scopus; AS: number of articles Scopus; IFW: impact factor WoS; CW: number of citations WoS; TC/A W: total citations per article WoS; h-i W: Hirsch index WoS.

**Table 3:** Articles with more citations in WoS.

	Article Title	Author	Journal	IF	TC
1	Golf injuries—An overview	Theriault, G.; Lachance, P.	Sport Medicine	11.14	108
2	‘We haven’t got a seat on the bus for you’ or ‘all the seats are mine’: narratives and career transition in professional golf	Carless, D.; Douglas, K.	Qualitative Research in Sport Exercise and Health	6.73	88
3	Why older Australians participate in exercise and sport	Kolt, G.S.; Driver, R.P.; Giles, L.C.	Journal of Aging and Physical Activity	1.96	88
4	Prevalence and correlates of participation in fall prevention exercise/physical activity by older adults	Merom, D.; Pye, V.; Macniven, R.; et al.	Preventative Medicine	4.01	87
5	Clinical outcome and return to sport after the surgical treatment of spondylolysis in young athletes	Debnath, U.K.; Freeman, B.J.C.; Gregory, P.; et al.	Journal of Bone and Joint Surgery—British Volume	3.30	82

IF: Impact factor; TC total citations per article.

**Table 4:** Most relevant affiliations (Scopus and WoS).

R	Affiliation Scopus	AS	Affiliation WoS	AW	Citations WoS	TC/Art WoS	h-Index WoS
1	The University of Edinburgh	5	University of Sydney	4	145	36, 25	4
2	University of Alberta	5	University of Alberta	4	50	12, 50	3
3	University of North Carolina	4	University of North Carolina	4	19	4, 75	3
4	Kyung Hee University	3					
5	Royal Infirmary of Edinburgh	3					
6	Hong Kong Polytechnic University	3	University of Edinburgh	4	6	1, 50	1

R: Ranking; AS: number of articles Scopus; AW: number of articles WoS; TC/Art: total citations per article; h-Index: Hirsch index.

**Table 5:** Most relevant financial support entities.

R	Financial Support Entities	A	C	TC/Art	h-Index
1	National Health and Medical Research Council of Australia	4	110	27.50	3
2	National Institutes of Health NIH USA	3	11	3.67	1
3	United States Department of Health and Human Services	3	11	3.67	1
4	Australian Government	2	7	3.50	1
5	Department of Industry Innovation and Science (Australia)	2	7	3.50	1
6	NIH National Heart Lung Blood Institute NHLBI (USA)	2	0	0.00	0

R: Ranking; A: number of articles; C: number of citations; TC/A: total citations per article; h-Index: Hirsch index.

**Table 6:** Most relevant keywords.

Keyword	Occurrences	Total Link Strength
golf	22	19
health	21	21
exercise	19	19
physical-activity	18	17
sport	17	16
physical activity	12	12
walking	11	11
injuries	10	9
performance	9	8
sports	9	9
adults	7	7
participation	7	7
prevention	6	6
rehabilitation	6	6
cognition	5	5
disease	5	5
men	5	5
mortality	5	5

## Supplementary Materials

The following supporting information can be downloaded at: https://www.mdpi.com/article/10.3390/healthcare10071322/s1, Table S1: Authors by numbers of publications WoS and Scopus.
