# Supplementary material for: Golf and Health, More than 18 Holes—A Bibliometric Analysis
Source: Healthcare (Basel). 2022 Jul 16;10(7):1322. doi: 10.3390/healthcare10071322 (PMC9317188; doi:10.3390/healthcare10071322)
Supplement: Supplementary file 1 [file healthcare-10-01322-s001.zip › healthcare-1784476-supplementary.pdf]

Table. Authors by numbers of publications WoS and Scopus.

R	Author Scopus	Afiliation Scopus	Author WoS	Afiliation WoS	hi W	N.A. WoS	C W	TC/Art WoS	First/Last WoS
1	Murray, Andrew	Uni. of Edimburgh (UK)	Murray, Andrew	Uni. of Edimburgh (UK)	14	**4**	**6**	1,50	2020/2021
2	Bauer, Iris	Eberhard Karls Uni. of Tubingen (DEU)	Bauer, Iris	Eberhard Karls Uni. of Tubingen (DEU)	12	3	20	6,67	1998/2003
3	Bauer, Ulrich	Univ Appl Sci Berlin HTW (DEU)	Bauer, Ulrich	Univ Appl Sci Berlin HTW (DEU)	25	3	20	6,67	1998/2003
4	Buckley, Jonathan	Uni. of South Australia (AU)	Robinson, Patrick	Univ. Of London	14	3	5	1,67	2020/2021
5	Cameron, P.A.	Monash Uni. Melbourne, Australia	Benes, Ksenija	Royal Wolverhampton NHS Trust (UK)	4	2	20	10,00	1998/2000
6	Fradkin, A.J.	Bloomsburg Uni., Bloomsburg, USA	Breitbarth, T	Swinburne Uni. of Technology (AU)	4	2	55	27,50	2019/2020
7	Gabbe, B.J.	Monash Uni. Melbourne, AU	Buckley, Jonathan	Uni. of South Australia (AU)	57	2	27	13,50	2016/2020
8	Hawkes, Roger	British Assoc Sport & Exercise Med (UK)	Bum, Chul-Ho	Kyung Hee University	4	2	74	37,00	2020/2021
9	Hui-Chan, C.W.Y.	Uni. of Illinois at Chicago, USA	Carless, David	University of Edimburgh (UK)	18	2	113	56,50	2009/2010
10	Mosewich, Amber D.	Uni. of Alberta (Canada)	Choi, Chulwan	Gachon University	4	2	74	37,00	2020/2021
11	Mutrie, N.	The Uni. of Edinburgh, UK	Close, Graeme	Liverpool John Moores University	4	2	33	16,50	2021
12	Robinson, P.G.	Royal Infirmary of Edinburgh, UK	Courneya, Kerry	University of Alberta	88	2	23	11,50	2013
13	Tsang, W.W.N.	Hong Kong Metropolitan Uni.	Douglas, Kitrina	Leeds Beckett University	16	2	114	57,00	2009/2010
14	Unverdormen, Mrtin	Daiichi Sakyo	Evenson, Kelly	University of North Carolina (USA)	71	2	5	2,50	2019/2020
15	Archibald	Uni. of Dundee, Dundee, UK	Freeman, Paul	University of Essex	19	2	56	28,00	2010/2021
16	Benes, Ksenija	Royal Wolverhampton NHS Trust (UK)	Hawkes, Roger	British Assoc Sport & Exercise Med (UK)	10	2	5	2,50	2020/2020
17	Breitbarth	Swinburne Uni. of Technology (AU)	Huth, Christop.	Bundeswehr Uni. Munich (Germany)	5	2	55	27,50	2019/2020
18	Bum, Chul-Ho	Kyung Hee Uni.	Kinane, Denis	University of Bern	60	2	32	16,00	2021
19	Choi, Chulwan	Gachon Uni.	Kolb, Meike	University of Munster	5	2	16	8,00	2000/2003
20	Close, Graeme	Liverpool John Moores Uni.	Meron, Dafna	Western Sydney University (Australia)	32	2	95	47,50	2012/2014
21	Courneya, Kerry	Uni. of Alberta	Mosewich, Amber D.	University of Alberta (Canada)	11	2	27	13,50	2016/2020
22	Evenson, Kelly	Uni.of North Carolina (USA)	Nowacki, PE	Justus Liebig Uni. Giessen (DEU)	8	2	16	8,00	2000/2003
23	Grant, Liz	The Uni. of Edinburgh, U K	Porter, Anna K	University of North Carolina (USA)	4	2	5	2,50	2019/2020
24	Huth, Christop	Bundeswehr UnI. Munich (DEU)	Stenner, Brad J.	University of South Australia (Australia)	3	2	27	13,50	2016/2020
25	Kelly, Paul	The University of Edinburgh, UK	Stude, Davd E.	SW Chiropract LLC (USA)	4	2	6	3,00	2001/2008
26	Kinane, Denis	University of Bern	Unverdormen	Daiichi Sakyo	17	2	43	21,50	1998/2003
27	Kolb, Meike	University of Munster	Vallbracht, Christian	Herz & Kreislaufzentrum (Germany)	17	2	20	10,00	1998/2000

R: Ranking; hi W: Hirsch index Wos; N.A.W: Number of citations; CW: Number of citations Wos; TC/Art W: Total citations per Article Wos.
